# Supplementary material for: PEAK1 maintains tight junctions in intestinal epithelial cells and resists colitis by inhibiting autophagy-mediated ZO-1 degradation
Source: Nat Commun. 2025 Jul 24;16:6777. doi: 10.1038/s41467-025-62107-z (PMC12290104; doi:10.1038/s41467-025-62107-z)
Supplement: Supplementary file 1 — Supplementary Information [file 41467_2025_62107_MOESM1_ESM.pdf]

Supplementary Information

PEAK1 maintains tight junctions in intestinal epithelial cells and resists colitis by inhibiting autophagy-mediated ZO-1 degradation

Zaikuan Zhang [1, 2, #], Yajun Xie [1, 3, #, *], Qiying Yi [4, #], Jianing Liu [5, 6, #], Lin Yang [1], Runzhi Wang [7], Jin Cai [7], Xinyi Li [1], Xiaosong Feng [1], Shixiang Yao [8], Zheng Pan [9], Magdalena Paolino [5, 6, *] and Qin Zhou [1, 7, *]

[1] The Ministry of Education Key Laboratory of Laboratory Medical Diagnostics, the College of Laboratory Medicine, Chongqing Medical University, Chongqing 400016, P.R. China.

[2] Chongqing University Three Gorges Hospital, Chongqing University, Chongqing 404100, P.R. China

[3] Western Institute of Digital-Intelligent Medicine, Chongqing 401329, P.R. China.

[4] The Experimental Animal Center, Chongqing Medical University, Chongqing 400016, P.R. China.

[5] Department of Medicine Solna, Center for Molecular Medicine, Karolinska Institutet, 171 77 Stockholm, Sweden.

[6] Karolinska University Hospital, 171 76, Stockholm, Sweden.

[7] The School of Basic Medical Sciences, Harbin Medical University, Harbin 150000, P.R. China.

[8] The College of Food Science, Southwest University, Chongqing 400715, P.R. China

[9] The College of Traditional Chinese Medicine, Chongqing Medical University, Chongqing 400016, P.R. China.

[#] These authors contributed equally: Zaikuan Zhang, Yajun Xie, Qiying Yi, Jianing Liu

[*] These authors are co-corresponding authors, and Yajun Xie is the lead contact author for this paper.

Correspondence: yjxie@cqmu.edu.cn; magdalena.paolino@ki.se; zhouqin@hrbmu.edu.cn.

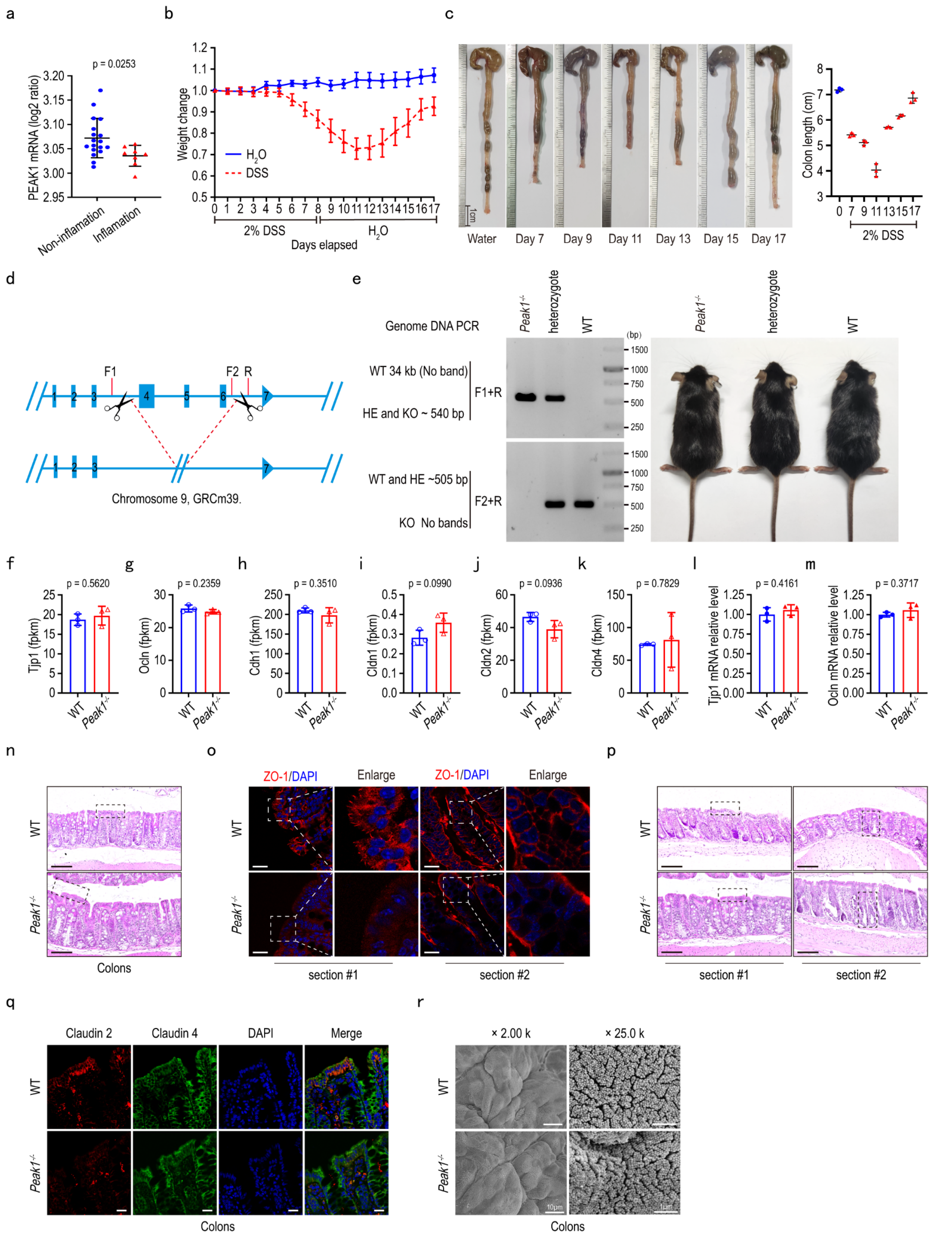

Supplementary Figure 1. PEAK1 deficiency disrupts the structure of intestinal epithelial cell tight junction in vivo.

(**a**) Analysis of PEAK1 mRNA expression in colonic mucosa samples of patients with or without signs of ulcerative colitis inflammation (analysis from the GEO database -GDS3119-). Data are

shown as mean ± SD. Non-inflammation group, n = 18; inflammation group, n = 8. Unpaired two-tailed Student's t-test. (**b**) Changes in body weight over time in C57BL/6 wild type (WT) mice during a colitis model. Mice were treated with 2% DSS for 8 days followed by 9 days of drinking water. Data are presented as a percentage of the initial body weight (mean ± SD). n = 5 in the water group. n = 20 initial animals in DSS group, and 3 mice at each specific day were taken for analysis shown in c. (**c**) Representative photographs of colons (left panel) and colon length measurements (right panel, mean ± SD) taken at the indicated time during the colitis model. n = 3 mice per group. (**d**) Schematic diagram showing the targeting strategy used to generate *Peak1* knockout mice via CRISPR/Cas9. The location of the primers used for genotyping (F1, F2, and R) are indicated. (**e**) Genotyping results of *Peak1* homozygous knockout (*Peak1*$^{-/-}$), heterozygous (*Peak1*$^{+/-}$), and wild-type (*Peak1*$^{+/+}$) mice (left panel). Representative photographs (right panel) show no overt phenotypic differences among the genotypes. *Peak1* knockout mice were generated by breeding heterozygous mice, with *Peak1*$^{+/+}$ (WT) littermates serving as controls. (**f-k**) RNA sequencing results of WT and *Peak1*$^{-/-}$ colons, showing no significant changes in *Tjp1* (f), *Ocln* (g), *Cdh1* (h), *Cldn1* (i), *Cldn2* (j), *Cldn4* (k). Data are shown as mean ± SD. n = 3 mice per group. Unpaired two-tailed Student's t-test. (**l-m**) qRT-PCR analysis of *Tjp1* (l) and *Ocln* (m) mRNA levels in colons from WT and *Peak1*$^{-/-}$ mice, with 18S as an internal control. Data are shown as mean ± SD for three technical replicates. *Tjp1,* gene codifying ZO-1; *Ocln*, gene codifying Occludin. Unpaired two-sided Student's t-test. (**n**) H&E staining of colon tissues. The black dashed line box indicates the region selected for the immunofluorescence imaging shown in Figure 1e. Scale bars, 100 μm. (**o-p**) Representative immunofluorescence images (o, scale bars, 20 μm) and corresponding H&E-stained images (p, scale bars, 100 μm) from two different sections of the WT and *Peak1*$^{-/-}$ colon tissues. Black dashed line

box in (p) represents the location of immunofluorescence in (o). (**q**) Representative immunofluorescence images showing Claudin 2 (red) and Claudin 4 (green) expression in WT and *Peak1*$^{-/-}$ colons. DAPI was used for nuclear counterstaining (blue). Scale bars, 20 μm. (**r**) Scanning electron microscopy (SEM) images showing microvilli in colons from WT and *Peak1*$^{-/-}$ mice. Left panel, 2000× magnification. scale bars, 10 μm. Right panel, 25000× magnification, scale bars, 1 μm. All experiments were repeated three times, yielding similar results.

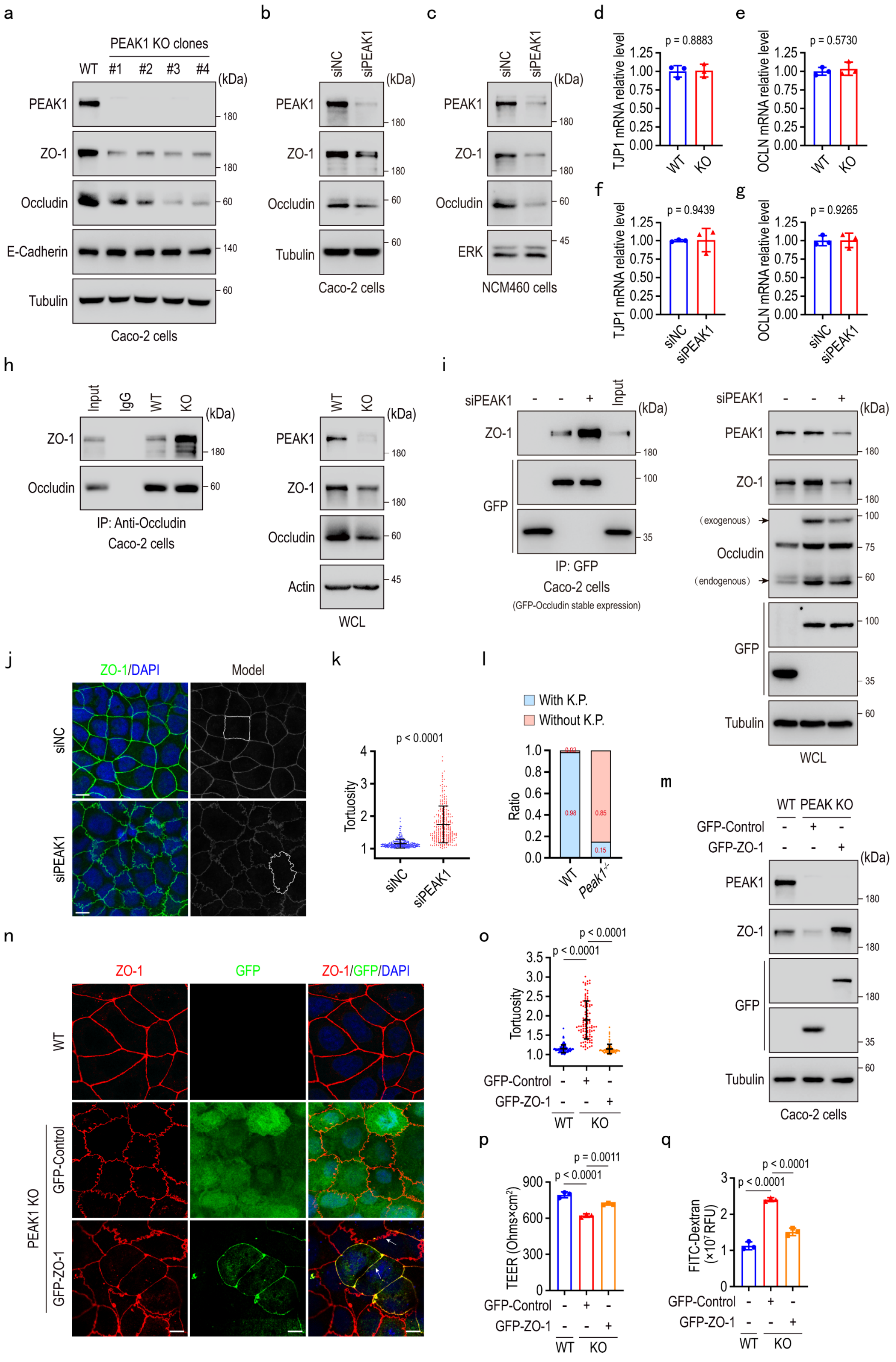
a
PEAK1 KO clones
WT #1 #2 #3 #4 (kDa)
PEAK1
180
ZO-1
180
Occludin
60
E-Cadherin
140
Tubulin
60
Caco-2 cells
b
siNC siPEAK1 (kDa)
PEAK1
180
ZO-1
180
Occludin
60
Tubulin
60
Caco-2 cells
c
siNC siPEAK1 (kDa)
PEAK1
180
ZO-1
180
Occludin
60
ERK
45
NCM460 cells
d
p = 0.8883
TJP1 mRNA relative level
WT KO
e
p = 0.5730
OCLN mRNA relative level
WT KO
f
p = 0.9439
TJP1 mRNA relative level
siNC siPEAK1
g
p = 0.9265
OCLN mRNA relative level
siNC siPEAK1
h
Input IgG WT KO (kDa)
ZO-1
180
Occludin
60
IP: Anti-Occludin
Caco-2 cells
WT KO (kDa)
PEAK1
180
ZO-1
180
Occludin
60
Actin
45
WCL
i
siPEAK1 - - + Input (kDa)
ZO-1
180
GFP
100
35
IP: GFP
Caco-2 cells
(GFP-Occludin stable expression)
siPEAK1 - - + (kDa)
PEAK1
180
ZO-1
180
(exogenous)
Occludin
(endogenous)
100
75
60
GFP
100
35
Tubulin
60
WCL
j
ZO-1/DAPI
Model
siNC
siPEAK1
k
p < 0.0001
Tortuosity
siNC siPEAK1
l
With K.P.
Without K.P.
Ratio
0.02
0.98
0.85
0.15
WT Peak1-/-
m
WT PEAK KO
GFP-Control - + -
GFP-ZO-1 - - +
(kDa)
PEAK1
180
ZO-1
180
GFP
180
35
Tubulin
60
Caco-2 cells
n
ZO-1
GFP
ZO-1/GFP/DAPI
WT
PEAK1 KO
GFP-Control
GFP-ZO-1
o
p < 0.0001
p < 0.0001
Tortuosity
GFP-Control - + -
GFP-ZO-1 - - +
WT KO
p
p = 0.0011
p < 0.0001
TEER (Ohms×cm²)
GFP-Control - + -
GFP-ZO-1 - - +
WT KO
q
p < 0.0001
p < 0.0001
FITC-Dextran (×10⁷ RFU)
GFP-Control - + -
GFP-ZO-1 - - +
WT KO

Supplementary Figure 2. PEAK1 deletion disrupts the arrangement of tight junctions in vitro. (**a)** Western blot analysis of PEAK1, ZO-1, and Occludin expression in four different PEAK1 knockout Caco-2 monoclonal cell lines. (**b-c**) Western blot analysis of PEAK1, ZO-1 and Occludin expression in Caco-2 (b) and NCM460 cells (c) transfected with either control siRNA (siNC) or siRNA targeting PEAK1 (siPEAK1). (**d-g**) qRT-PCR analysis of *TJP1* (d, f) and *OCLN* (e, g) mRNA levels in WT and PEAK1 knockout or knockdown Caco-2 cells, with 18S as internal control. Data are shown as mean ± SD for three technical replicates. Unpaired two-tailed Student's t-test. (**h**) Co-immunoprecipitation using anti-Occludin antibodies in protein lysates from PEAK1 WT and PEAK1 KO Caco-2 cells, showing higher ZO-1/Occludin binding in the absence of PEAK1. Whole-cell lysates (WCL) served as input control. (**i**) Caco-2 cells stably expressing GFP-tagged Occludin were transfected with siRNAs targeting PEAK1 (siPEAK1) or non-targeting control siRNA (siNC). Cellular lysates were collected 48 hours post-transfection for immunoprecipitation with anti-GFP magnetic beads. (**j**) Representative immunofluorescence images for ZO-1 (green) in Caco-2 cells transfected with siRNA targeting PEAK1 (siPEAK1) or non-targeting controls (siNC). (**k**) Quantification of barrier tortuosity, determined using ZO-1 immunofluorescence. Scale bars, 10 μm. Data are presented as mean ± SD. siNC, n = 250 cells; siPEAK1, n = 273 cells. Unpaired two-tailed Mann-Whitney test. (**l**) The percent analysis of tight junctions with kissing points between two adjacent intestinal epithelial cells observed by transmission electron microscope, 6 mice were analyzed in each group, and 10 tight junctions in the colon epithelial site were observed in each mouse, K.P. = Kissing Point. (**m**) Western blot analysis in WT or PEAK1 knockout (KO) Caco-2 cells overexpressing GFP-Control and GFP-tagged ZO-1. (**n-o**) Representative confocal images (n) and barrier tortuosity quantification (o) in WT and PEAK1 knockout (KO) Caco-2 cells transfected

with GFP-tagged ZO-1 or GFP-Control plasmids. Scale bars, 10 μm. Data are shown as mean ± SD. n = 100 cells per group. Kruskal-Wallis test, followed by Dunn's multiple comparisons test. (**p-q**) TEER (p) and fluorescence intensity of FITC-dextran in the lower chambers of trans-well inserts (0.4 μm) seeded with WT or PEAK1 KO stably expressing GFP-Control and GFP-tagged ZO-1 Caco-2 cell monolayers (q). Data are expressed as mean ± SD for three biological replicates. One-way ANOVA, followed by Tukey's multiple comparisons test. All experiments were repeated three times, yielding similar results.

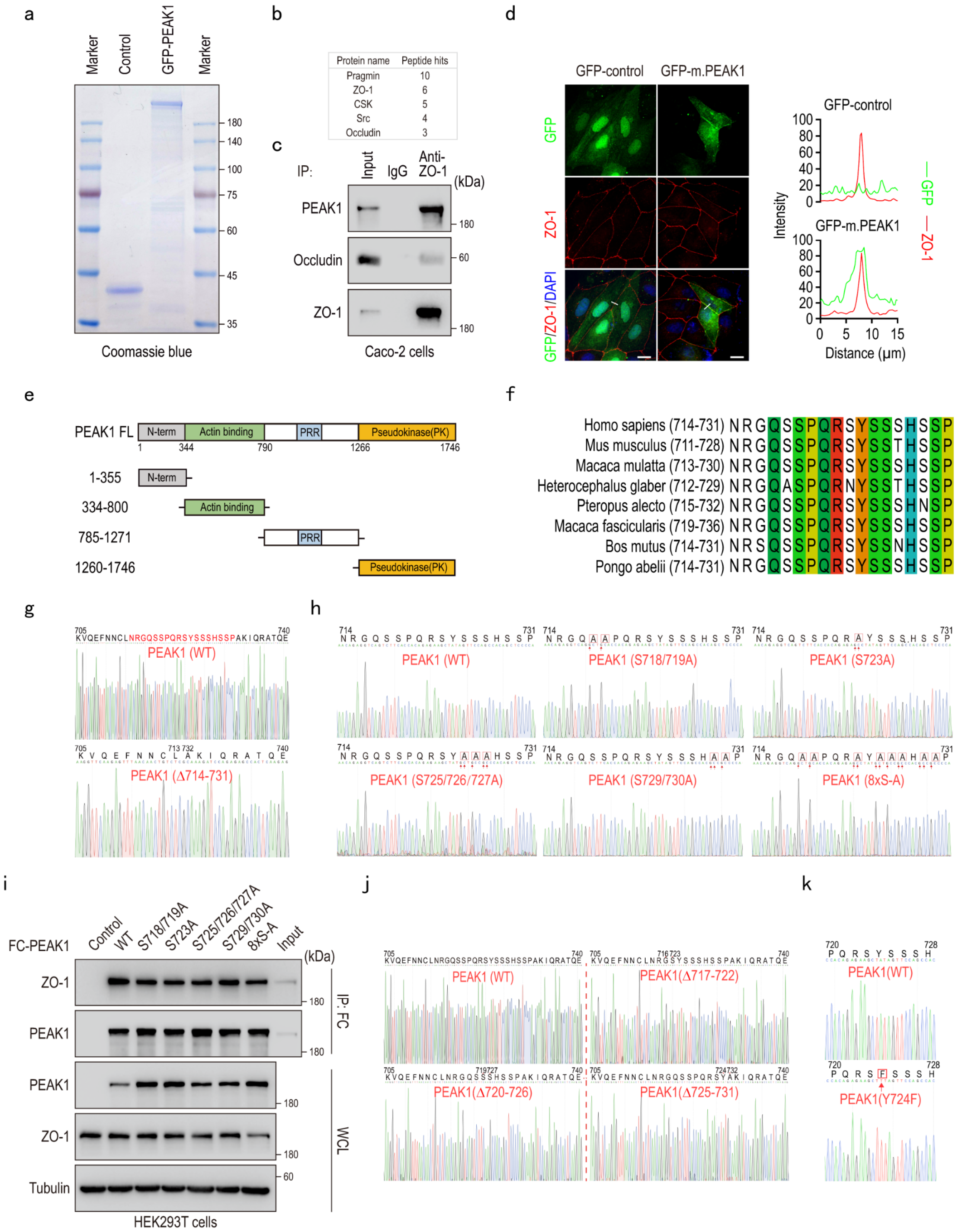

Supplementary Figure 3. Phosphorylation of PEAK1 on Y724 influences its association with ZO-1.

(**a**) Coomassie Brilliant Blue-stained gel showing proteins immunoprecipitated with anti-GFP magnetic beads from the lysates of HEK293T cells transfected GFP-Control or GFP-tagged PEAK1.

(**b**) Table listing the PEAK1-interacting proteins identified by mass spectrometry. (**c**) Immunoprecipitation analysis using anti-ZO1 antibodies in Caco-2 cells confirming the interaction between PEAK1, ZO-1, and Occludin. (**d**) Representative immunofluorescence images (left panel) for ZO-1 (red) and GFP-tag (green) of Madin-Darby Canine Kidney (MDCK) cells transfected with either a GFP-Control or GFP-tagged murine PEAK1. Right panel: co-localization analysis of GFP and ZO-1. Scale bars, 25 µm. (**e**) Schematic diagram of the full-length (FL) PEAK1 and its truncated variants. PRR: proline-rich region. (**f**) Conservation analysis of the 714-731 amino acid region of PEAK1 across different species, conducted using an online tool (https://www.rcsb.org/). (**g**) Sequencing results showing the PEAK1 full-length sequence (upper panel) and sequence amino acids 714-731 deleted (lower panel). The red amino acid sequence denotes amino acids 714-731. (**h**) Sequencing results of diverse PEAK1 mutants, confirming the targeted modifications. The mutated amino acids are highlighted in a red solid box and the mutated bases are indicated by a red arrow. (**i**) Immunoprecipitation of PEAK1 in HEK293T cells transfected with plasmids for the expression of FC-tagged PEAK1 wildtype (WT) or diverse FC-tagged PEAK1 mutants. Western blot for ZO-1 was used to map the relevant PEAK1 regions that interact with ZO-1. (**j-k**) Sequencing results for diverse PEAK1 deletions (j) as well as PEAK1 WT (k, upper panel) and Y724F mutant (k, lower panel) confirming the intended mutations. The red solid box represents the mutated amino acid, and the red arrow indicates the mutated bases. All experiments were repeated three times, yielding similar results.

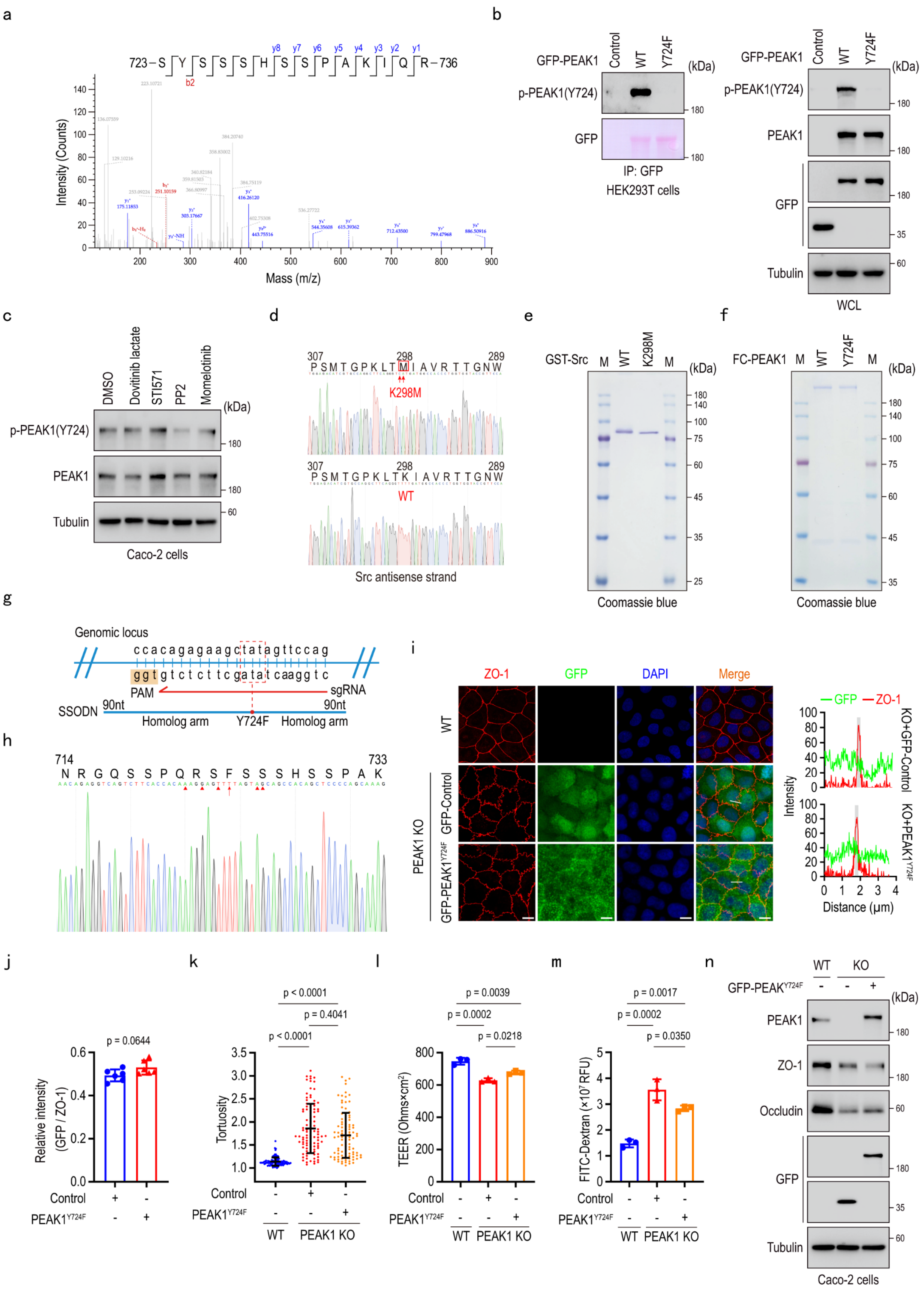

Supplementary Figure 4. Src-phosphorylated PEAK1 Y724 contributes to tight junction homeostasis.

(**a**) Total phosphorylated sites on PEAK1 identified by mass spectrometry. HEK293T cells were

transfected with GFP-tagged PEAK1 plasmids. After lyses, PEAK1 was immunoprecipitated with anti-GFP magnetic beads, followed by SDS-PAGE and MS analysis. (**b**) HEK293T cells transfected with GFP-Control (Control), GFP-tagged PEAK1 (WT), and GFP-tagged PEAK1^{Y724F} mutant (Y724F) plasmids were starved without serum for 12 hours and then stimulated with fetal bovine serum (FBS) for another 0.5 hour. PEAK1 proteins were immunoprecipitated with anti-GFP magnetic beads, and levels of Y724 phosphorylation in PEAK1^{WT} and PEAK1^{Y724F} mutant were detected using the p-PEAK1 (Y724) antibody. GFP-tagged PEAK1 proteins were visualized using Ponceau staining. (**c**) Western blot analysis of PEAK1 phosphorylated status in Caco-2 cells treated with various kinase inhibitors for 2 hours (Dovitinib lactate, 30 nM; STI571 Mesylate, 100 nM; PP2, 10 μM; Momelotinib, 20 nM). (**d**) Sequencing results confirming the generation of the Src^{K298M} point mutant (upper panel). The lower panel shows the control sequence of Src^{WT}. The red solid box highlights the mutated amino acid, and the red arrow indicates the mutated bases. (**e**-**f**) Coomassie Brilliant Blue-stained gels for the purified GST-tagged Src^{WT} and GST-tagged Src^{K298M} (e), and FC-tagged PEAK1^{WT} and FC-tagged PEAK1^{Y724F} proteins (f). (**g**) Schematic diagram of the knock-in strategy for introducing the PEAK1^{Y724F} mutation in Caco-2 cells using CRISPR/Cas9. Single-stranded DNA oligonucleotides (ssODNs) with 90-nt homolog arms flanked the point mutation (Y724F). The red line indicates sgRNA. (**h**) Genome sequencing results confirming the successful CRISPR/Cas9-mediated generation of Caco-2 cells carrying the Y724F in situ mutation. The red triangle indicates the synonymous mutation base site, and the red arrow represents the point mutation site. (**i**) Representative immunofluorescence images (left panel) for ZO-1 (red) and GFP-tag (green) of *PEAK1*$^{-/-}$ Caco-2 cells transfected with either a GFP-Control or GFP-tagged PEAK1^{Y724F}. Right panel: co-localization analysis of GFP and ZO-1. Scale bars, 10 μm. (**j**)

Relatively quantification of GFP and ZO-1 fluorescence intensity in the gray areas showing in i. Data are shown as mean ± SD. n = 6 independent samples per group. Unpaired two-sided Student's t-test. (**k**) Quantification of barrier tortuosity for i. Data are presented as mean ± SD. n = 100 cells per group. Kruskal-Wallis test, followed by Dunn's multiple comparisons test. (**l-m**) TEER (l) and fluorescence intensity of FITC-dextran in the lower chambers of trans-well inserts (0.4 μm) seeded with WT or PEAK1 KO expressing GFP-Control and GFP-tagged PEAK1^{Y724F} Caco-2 cell monolayers (m). Data are expressed as mean ± SD for three biological replicates. One-way ANOVA, followed by Tukey's multiple comparisons test. (**n**) Western blot analysis of ZO-1 and Occludin expression in WT, PEAK1 knockout (KO), and PEAK1 KO Caco-2 cells overexpressing GFP-tagged PEAK1^{Y724F}. All experiments were repeated three times, yielding similar results.

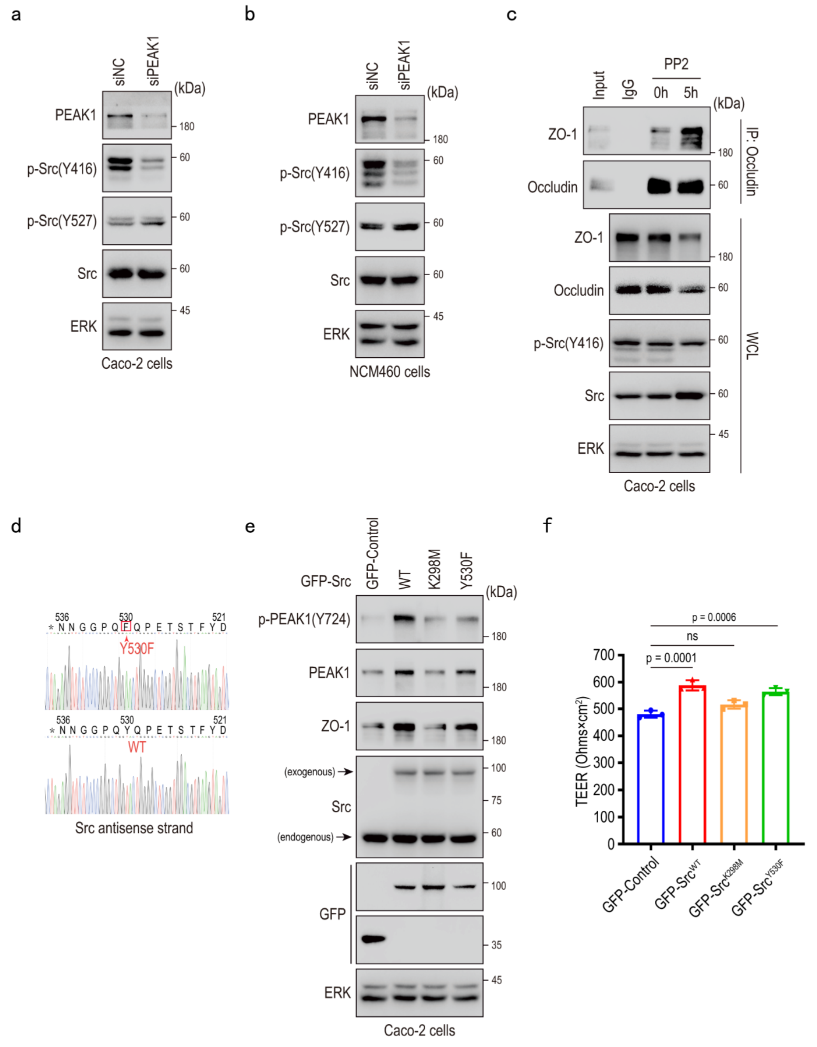

Supplementary Figure 5. PEAK1-mediated remodeling of tight junctions depends on Src activity.

(**a-b**) Western blot analysis of p-Src^{Y416} (indicates Src active state) and p-Src^{Y527} (indicates Src inactive state) in Caco-2 cells (a) and NCM460 cells (b) after transfected with either control siRNAs (siNC) or siRNA targeting PEAK1 (siPEAK1). Cells were lysed 48 hours post-transfection, and lysates were analyzed using the specified antibodies. (**c**) Co-immunoprecipitation with anti-Occludin antibodies in Caco-2 cells treated with 10 μM PP2 inhibitor for 5 hours, followed by western blotting using indicated antibodies. (**d**) Sequencing results confirming the Src^{Y530F} point

mutation (upper panel). The lower panel shows sequencing of Src^{WT} as a control. The red solid box indicates the mutated amino acid, and the red arrow denotes the mutated bases. (**e**) Western blot analysis of ZO-1 and phosphorylation of PEAK1 Y724 in Caco-2 cells expressing Src^{WT}, Src^{K298M} (kinase-inactive Src), and Src^{Y530F} (kinase-active Src). (**f**) Transepithelial electrical resistance (TEER) in Caco-2 cells expressing GFP-Control (GFP), Src^{WT}, Src^{K298M} (kinase-inactive Src) and Src^{Y530F} (kinase-active Src) vectors. Data are presented as mean ± SD, n = 3 biological replicates per group. One-way ANOVA, followed by Tukey's multiple comparisons test. All experiments were repeated three times, yielding similar results.

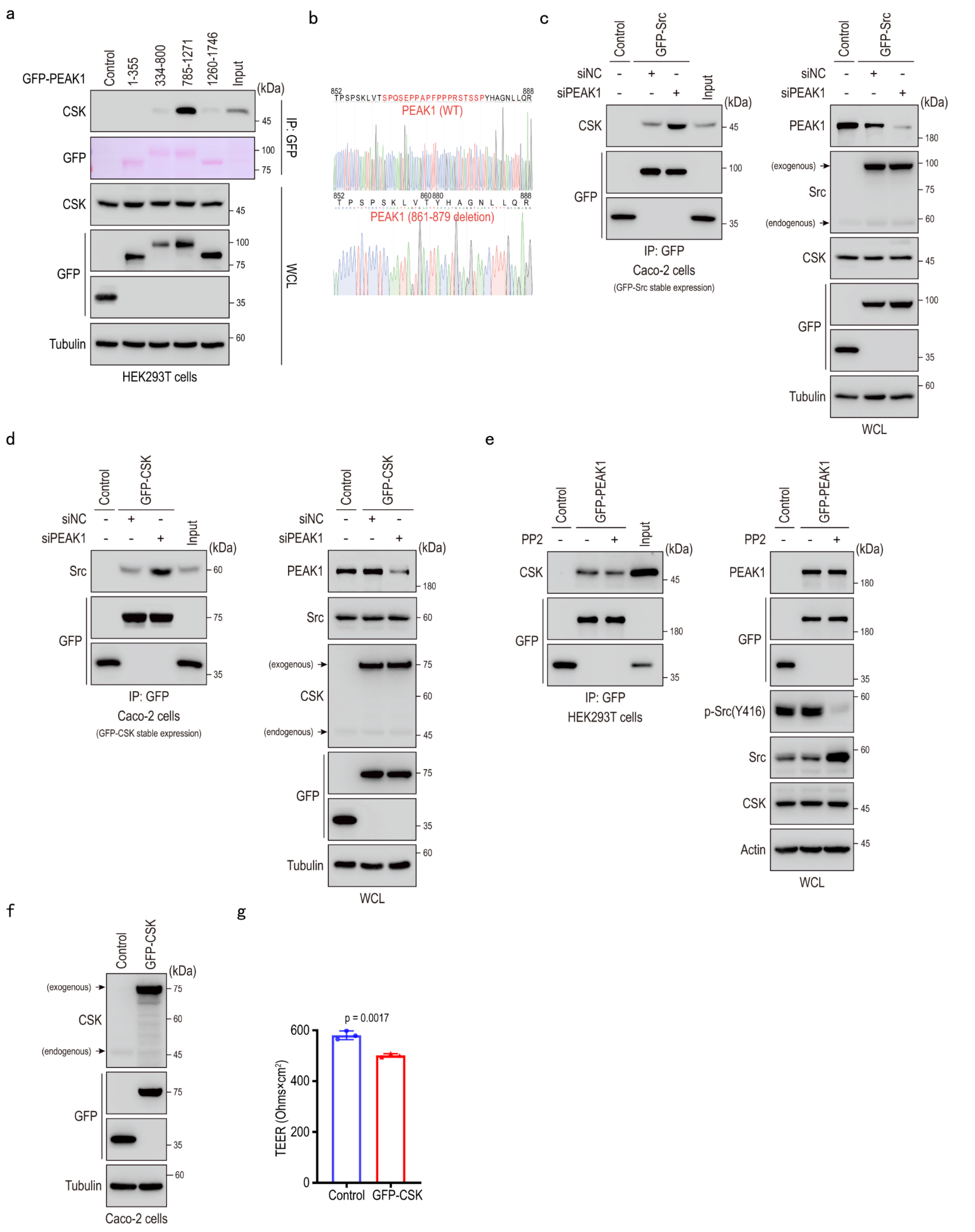

Supplementary Figure 6. PEAK1 modulates Src activity by scaffolding CSK.

(**a**) Immunoprecipitation using anti-GFP magnetic beads from HEK293T lysates overexpressing GFP-tagged PEAK1 truncations to map the PEAK1 region that binds CSK. Overexpressed GFP-tagged PEAK1 truncations were visualized with Ponceau staining. (**b**) Sequencing results

confirming the deletion of amino acids 861-879 (lower panel) in PEAK1. The sequence for wild-type PEAK1 is shown in the upper panel. The red amino acid sequence represents amino acids 861-879. (**c-d)** Co-immunoprecipitation analysis in Caco-2 cells to assess interactions between CSK and Src after PEAK1 knockdown with siRNA. (c) Src immunoprecipitation using anti-GFP magnetic beads in Caco-2 cells stably expressing GFP-tagged Src, treated with either non-targeting control siRNAs (siNC) or siRNAs targeting PEAK1 (siPEAK1). (d) CSK immunoprecipitation using anti-GFP magnetic beads in Caco-2 cells stably expressing GFP-tagged CSK and treated with either non-targeting control siRNAs (siNC) or siRNAs targeting PEAK1 (siPEAK1). Immunoprecipitation was followed by western blotting with the indicated antibodies. Whole-cell lysates (WCL) are shown as input controls. (**e**) HEK293T cells were transfected with GFP-Control (Control) or GFP-tagged PEAK1. After 48 hours, cells were treated with DMSO or 10 μM PP2 inhibitor for 2 hours. Cell lysates were then subjected to immunoprecipitation using anti-GFP magnetic beads and analyzed by western blotting with the indicated antibodies. (**f**) Western blot confirming CSK expression in the stably GFP-tagged CSK expressing Caco-2 cells. (**g**) TEER measurements in Caco-2 cells stably expressing GFP-Control (Control) or GFP-tagged CSK. Data are presented as mean ± SD for three biological replicates. Unpaired two-sided Student's t-test. All experiments were repeated three times, yielding similar results.

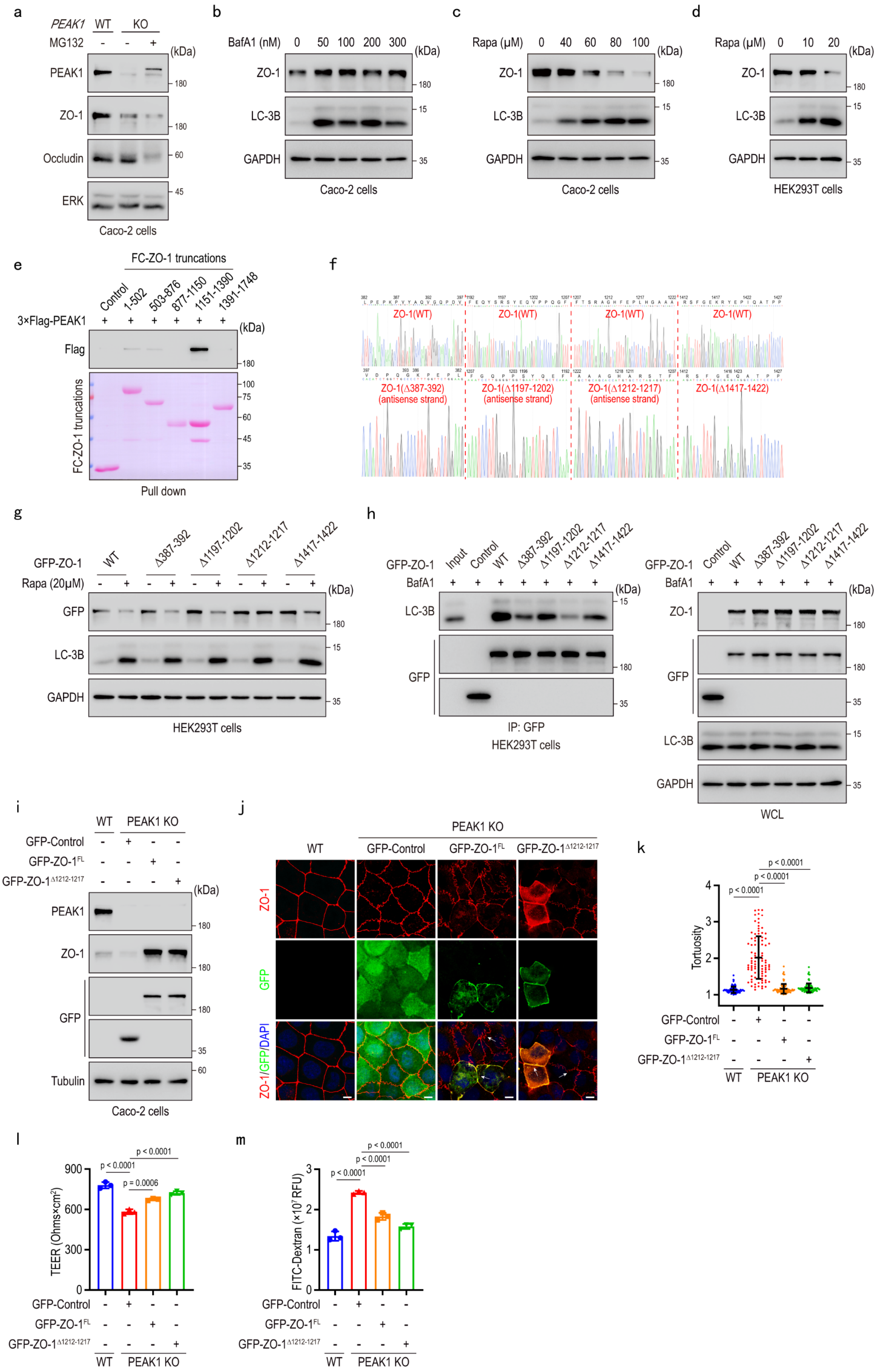

a
PEAK1 WT KO
MG132 - - +
(kDa)
PEAK1
180
ZO-1
180
Occludin
60
ERK
45
Caco-2 cells
b
BafA1 (nM) 0 50 100 200 300
(kDa)
ZO-1
180
LC-3B
15
GAPDH
35
Caco-2 cells
c
Rapa (μM) 0 40 60 80 100
(kDa)
ZO-1
180
LC-3B
15
GAPDH
35
Caco-2 cells
d
Rapa (μM) 0 10 20
(kDa)
ZO-1
180
LC-3B
15
GAPDH
35
HEK293T cells
e
FC-ZO-1 truncations
Control 1-502 503-876 877-1150 1151-1390 1391-1748
3×Flag-PEAK1 + + + + + +
(kDa)
Flag
180
FC-ZO-1 truncations
100
75
60
45
35
Pull down
f
ZO-1(WT)
ZO-1(WT)
ZO-1(WT)
ZO-1(WT)
ZO-1(Δ387-392) (antisense strand)
ZO-1(Δ1197-1202) (antisense strand)
ZO-1(Δ1212-1217) (antisense strand)
ZO-1(Δ1417-1422)
g
GFP-ZO-1 WT Δ387-392 Δ1197-1202 Δ1212-1217 Δ1417-1422
Rapa (20μM) - + - + - + - + - +
(kDa)
GFP
180
LC-3B
15
GAPDH
35
HEK293T cells
h
GFP-ZO-1 Input Control WT Δ387-392 Δ1197-1202 Δ1212-1217 Δ1417-1422
BafA1 + + + + + + +
(kDa)
LC-3B
15
GFP
180
35
IP: GFP
HEK293T cells
GFP-ZO-1 Control WT Δ387-392 Δ1197-1202 Δ1212-1217 Δ1417-1422
BafA1 + + + + + +
(kDa)
ZO-1
180
GFP
180
35
LC-3B
15
GAPDH
35
WCL
i
WT PEAK1 KO
GFP-Control - + - -
GFP-ZO-1FL - - + -
GFP-ZO-1Δ1212-1217 - - - +
(kDa)
PEAK1
180
ZO-1
180
GFP
180
35
Tubulin
60
Caco-2 cells
j
PEAK1 KO
WT GFP-Control GFP-ZO-1FL GFP-ZO-1Δ1212-1217
ZO-1
GFP
ZO-1/GFP/DAPI
k
p < 0.0001
p < 0.0001
p < 0.0001
Tortuosity
4
3
2
1
GFP-Control - + - -
GFP-ZO-1FL - - + -
GFP-ZO-1Δ1212-1217 - - - +
WT PEAK1 KO
l
p < 0.0001
p < 0.0001
p = 0.0006
TEER (Ohms×cm²)
900
600
300
0
GFP-Control - + - -
GFP-ZO-1FL - - + -
GFP-ZO-1Δ1212-1217 - - - +
WT PEAK1 KO
m
p < 0.0001
p < 0.0001
p < 0.0001
FITC-Dextran (×10⁷ RFU)
3
2
1
0
GFP-Control - + - -
GFP-ZO-1FL - - + -
GFP-ZO-1Δ1212-1217 - - - +
WT PEAK1 KO

Supplementary Figure 7. PEAK1 deletion triggers ZO-1 degradation by exposing an LC3-interacting region on ZO-1.

(**a**) Western blot analysis of ZO-1 levels in wild-type (WT) and *PEAK1* knockout (KO) Caco-2 cells treated for 12 hours with 10 μM MG132 (proteasome inhibitor). (**b**) Western blot analysis of ZO-1 levels in wild-type Caco-2 cells treated for 12 hours with gradient concentrations of bafilomycin A1. (**c-d**) Western blot analysis of ZO-1 levels in Caco-2 (c) and HEK293T (d) wild-type cells treated with a range of Rapamycin (Rapa, autophagy activator) concentrations for 12 hours (Caco-2) or 24 hours (HEK293T). (**e**) In vitro pull-down assay to detect interactions between FC-tagged ZO-1 truncations and 3×Flag-tagged PEAK1. PEAK1 was detected using an anti-flag antibody, and FC-tagged ZO-1 truncations were visualized with Ponceau staining. (**f**) Sequencing results confirming the sequences of ZO-1 full-length and various ZO-1 deletions. (**g**) Western blot analysis comparing the stability of ZO-1 full-length (WT) and several truncated forms cells upon autophagy induction. HEK293T cells were transfected with GFP-tagged ZO-1 constructs, and treated with 20 μM Rapamycin for 24 hours. (**h**) Immunoprecipitation of GFP-tagged ZO-1 full-length (WT) and several truncated forms in HEK293T treated with 100 nM BafA1 for 12 hours, used to map the critical ZO-1 region interacting with LC-3B. (**i**) Western blot analysis in WT or PEAK1 knockout (KO) Caco-2 cells overexpressing GFP-Control, GFP-tagged ZO-1^{FL}, and GFP-tagged ZO-1$^{\Delta 1212\text{-}1217}$. FL, full-length. (**j-k**) Representative confocal images (j) and barrier tortuosity quantification (k) in WT and PEAK1 knockout (KO) Caco-2 cells transfected with GFP-Control, GFP-tagged ZO-1^{FL} or GFP-tagged ZO-1$^{\Delta 1212\text{-}1217}$ plasmids. Scale bars, 10 μm. Data are shown as mean ± SD. n = 100 cells per group. Kruskal-Wallis test, followed by Dunn's multiple comparisons test. (**l-m**) TEER (l) and fluorescence intensity of FITC-dextran in the lower chambers of trans-well inserts (0.4 μm)

seeded with WT or PEAK1 KO stably expressing GFP-Control, GFP-tagged ZO-1 or GFP-tagged ZO-1$^{\Delta 1212\text{-}1217}$ Caco-2 cell monolayers (m). Data are expressed as mean ± SD. n = 3 biological replicates per group. One-way ANOVA, followed by Tukey's multiple comparisons test. All experiments were repeated three times, yielding similar results.

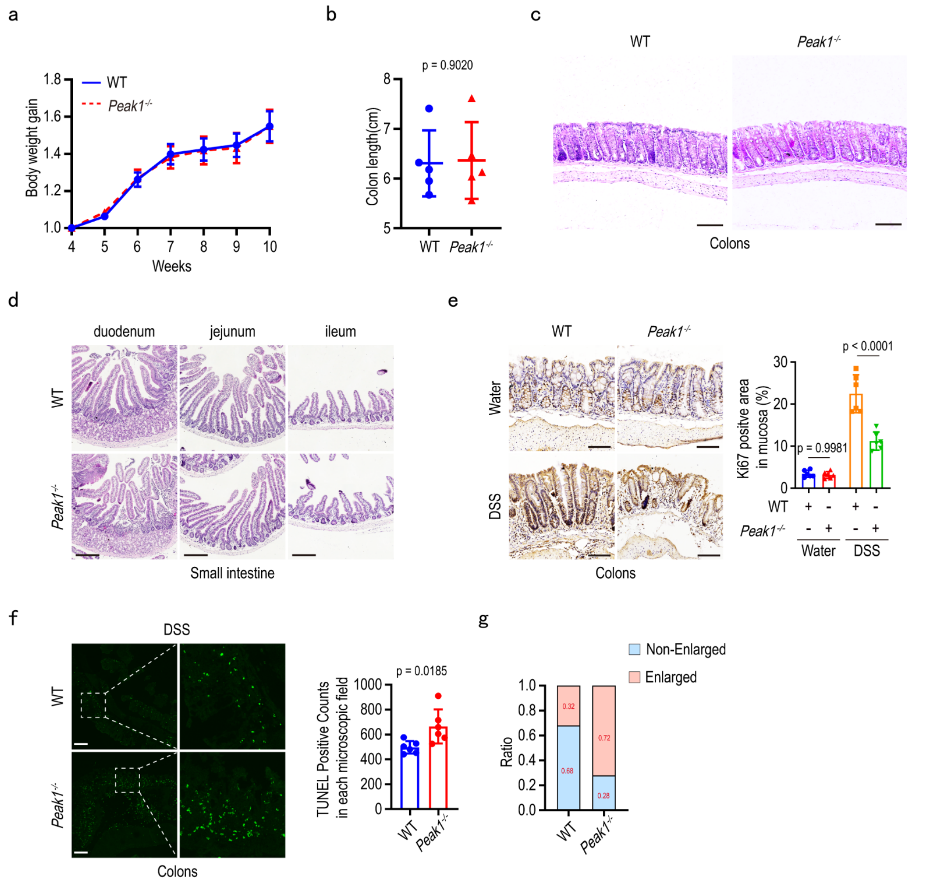

Supplementary Figure 8. ***Peak1*$^{-/-}$ unchallenged mice do not show signs of spontaneous intestinal inflammation.**

(**a**) Changes in body weight over time for both WT and *Peak1*$^{-/-}$ mice, expressed as a percentage of their initial body weight. n = 7 mice per group. Data are presented as mean ± SEM, p = 0.9902, Unpaired two-sided Student's t-test. (**b**) Measurements of colon length in unchallenged WT and *Peak1*$^{-/-}$ mice. Data are presented as mean ± SD. n = 5 mice per group. Unpaired two-sided Student's t-test. (**c**) Representative images of H&E-staining of distal colon cross-sections from unchallenged WT and *Peak1*$^{-/-}$ mice. Scale bars, 100 µm. No signs of spontaneous inflammation are observed in either group. (**d**) Representative images of H&E staining of duodenum, jejunum, and ileum from unchallenged WT and *Peak1*$^{-/-}$ mice. Scale bars, 200 µm. (**e**) Left panel: immunohistochemistry for

detection of Ki67 expression in colon cross-sections from WT and *Peak1*$^{-/-}$ mice treated with or without 2% DSS. Scale bars, 100 μm. Right panel: quantification of Ki67 positive areas in crypts of colon. Data are presented as mean ± SD. n = 6 mice per group. One-way ANOVA, followed by Tukey's multiple comparisons test. (**f**) Left panel: TUNEL assay of WT and *Peak1*$^{-/-}$ colons treated with 2% DSS. Scale bars, 100 μm. Right panel: quantification of TUNEL positive puncta in all observed colon tissues. White dotted box represents the enlarged areas to the right. Data are presented as mean ± SD. n = 6 biological replicates per group. Unpaired two-sided Student's t-test. (**g**) The percent analysis of tight junctions with enlarged gaps between two adjacent intestinal epithelial cells observed by transmission electron microscope, 6 mice challenged with 2% DSS water were analyzed in each group, and 10 tight junctions in the colon epithelial site were observed in each mouse, usually, enlarged defined as obvious gaps between two IEC cells.

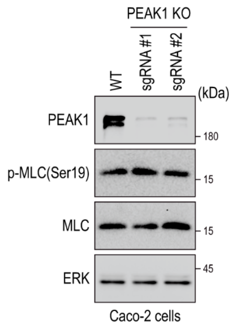

Supplementary Figure 9. PEAK1 deletion does not influence Myosin pathway.

Western blot analysis of myosin light chain 2 and phospho-myosin light chain 2 expression in different PEAK1 knockout Caco-2 cells. This experiment was repeated three times, yielding similar results.

Supplementary Table 1. Primers for recombinant DNA.

FC-PEAK1	Forward (5'-3'): cgcgtggatccccggaattcatgtctgcttgtaacacctttactg
	Reverse (5'-3'): cttcctctgccctcggtaccttaacggtgctgcagaattttcaca
GFP-PEAK1	Forward (5'-3'): aagggatccgaattcatgtctgcttgtaacacctttactg
	Reverse (5'-3'): atggtggtgctcgagttaacggtgctgcagaattttcaca
3×flag-PEAK1	Forward (5'-3'): aaaggatccgaattcatgtctgcttgtaacacctttactg
	Reverse (5'-3'): atggtggtgctcgagttaacggtgctgcagaattttcaca
FC-PEAK1 (1-355)	Forward (5'-3'): ggatccccggaattcatgtctgcttgtaacacctttactg
	Reverse (5'-3'): tgatggtggtgctcgagttactcagaacgtgattcttctgttaaa
FC-PEAK1 (334-800)	Forward (5'-3'): ggatccccggaattcatgcagagcatggtgtcatctgac
	Reverse (5'-3'): tgatggtggtgctcgagttaaggaatggcataaagctcttccaca
FC-PEAK1 (334-750)	Reverse (5'-3'): atggtggtgctcgagttactcctgagtgccttctattttggcc
FC-PEAK1 (334-700)	Reverse (5'-3'): atggtggtgctcgagttagcccctttttatgctctgtgctgttt
FC-PEAK1 (334-650)	Reverse (5'-3'): atggtggtgctcgagttattctccacttgttcccagaaaactt
FC-PEAK1 (334-600)	Reverse (5'-3'): atggtggtgctcgagttaattgttataactattaaatttaatt
FC-PEAK1 (785-1271)	Forward (5'-3'): ggatccccggaattcatgcaatctggccctaaagcttgcagtg
	Reverse (5'-3'): tgatggtggtgctcgagttacggcttctggatgcctcggccctgt
FC-PEAK1 (1260-1746)	Forward (5'-3'): ggatccccggaattcatgccctcttgcagacagggccgaggca
	Reverse (5'-3'): tggtgatggtggtgctcgagttaacggtgctgcagaattttcaca
PEAK1 (Δ714-731)	Forward (5'-3'): caagagtttaacaactgtctcgcaaagatccagaga
	Reverse (5'-3'): agtggctctctggatctttgcgagacagttgttaaa
PEAK1 (Δ717-722)	Forward (5'-3'): tgtctcaacagaggtagctatagttccagccacag
	Reverse (5'-3'): gctggaactatagctacctctgttgagacagttgt
PEAK1 (Δ720-726)	Forward (5'-3'): agaggtcagtcttcaagccacagctccccagcaaa
	Reverse (5'-3'): tggggagctgtggcttgaagactgacctctgttga
PEAK1 (Δ725-731)	Forward (5'-3'): ccacagagaagctatgcaaagatccagagagccact
	Reverse (5'-3'): tctctggatctttgcatagcttctctgtggtgaag
PEAK1 (Δ861-879)	Forward (5'-3'): ctctccctccaaattagtgacttaccatgcaggtaac
	Reverse (5'-3'): gcaaaaggttacctgcatggtaagtcactaatttgga
PEAK1 (Y724F)	Forward (5'-3'): gtcttcaccacagagaagctttagttccagccacag
	Reverse (5'-3'): gggagctgtggctggaactaaagcttctctgtggtg
PEAK1 (S718/719A)	Forward (5'-3'): ctcaacagaggtcaggctgcaccacagagaagctatagttc
	Reverse (5'-3'): atagcttctctgtggtgcagcctgacctctgttgagacagt
PEAK1 (S723A)	Forward (5'-3'): gtcagtcttcaccacagagagcctatagttccagcca
	Reverse (5'-3'): agctgtggctggaactataggctctctgtggtgaaga

PEAK1 (S725/726/727A)	Forward (5’-3’): ccacagagaagctatgctgccgcccacagctccccagcaaagat
	Reverse (5’-3’): tgctggggagctgtgggcggcagcatagcttctctgtggtgaag
PEAK1 (S729/730A)	Forward (5’-3’): tatagttccagccacgccgccccagcaaagatccagagagc
	Reverse (5’-3’): ctggatctttgctggggcggcgtggctggaactatagcttc
PEAK1 (8×S-A)	Forward (5’-3’): gctgcaccacagagagcctatgctgccgcccacgccgccccagcaaagatccagagagc
	Reverse (5’-3’): cggcgtgggcggcagcataggctctctgtggtgcagcctgacctctgttgagacagttg
FC-ZO-1	Forward (5’-3’): ggatccccggaattcatgtccgccagagctgcggc
	Reverse (5’-3’): cctctgccctcggtaccttaaaagtggtcaataagga
FC-ZO-1 (1-502)	Forward (5’-3’): ggatccccggaattcatgtccgccagagctgcggc
	Reverse (5’-3’): tctgccctcggtaccttacttctgagccaatatggtca
FC-ZO-1 (503-876)	Forward (5’-3’): ggatccccggaattcatgaagaaggatgtttatcgtcg
	Reverse (5’-3’): tctgccctcggtaccttaccgtgtaatggcagactccg
FC-ZO-1 (877-1150)	Forward (5’-3’): ggatccccggaattcatgtcctctgagcctgtaagaga
	Reverse (5’-3’): tctgccctcggtaccttaaggtgcctgttcgtaacgtg
FC-ZO-1 (1151-1390)	Forward (5’-3’): ggatccccggaattcatgagagcatccgccctgcggca
	Reverse (5’-3’): tctgccctcggtaccttactgagaatgcgctggctttg
FC-ZO-1 (1391-1748)	Forward (5’-3’): ggatccccggaattcatgaatcaatcaaatttttctag
	Reverse (5’-3’): tctgccctcggtaccttaaaagtggtcaataaggacag
FC-ZO-1 (Δ1-502)	Forward (5’-3’): ggatccccggaattcatgaagaaggatgtttatcgtcg
	Reverse (5’-3’): cctctgccctcggtaccttaaaagtggtcaataagga
FC-ZO-1 (Δ1151-1371)	Forward (5’-3’): tacgaacaggcacctcctgcacacattgccgccagc
	Reverse (5’-3’): aggtgcctgttcgtaacgtggtctgctgtcgtaaga
GFP-ZO-1 (Δ387-392)	Forward (5’-3’): cttctcttccagaaccaaaggggcaaccagatgtg
	Reverse (5’-3’): aaatccacatctggttgcccctttggttctggaag
GFP-ZO-1 (Δ1197-1202)	Forward (5’-3’): agtattttgagcaatattcaccaccccaaggattt
	Reverse (5’-3’): gaggtaaatccttggggtggtgaatattgctcaaa
GFP-ZO-1 (Δ1212-1217)	Forward (5’-3’): aaggatttacctctagagcacatggtgctgcagct
	Reverse (5’-3’): gggacagctgcagcaccatgtgctctagaggtaaa
GFP-ZO-1 (Δ1417-1422)	Forward (5’-3’): tggatagatcatttggcgagcaggccactccccct
	Reverse (5’-3’): ggaggagggggagtggcctgctcgccaaatgatct
GFP-Src	Forward (5’-3’): aagggatccgaattcatgggtagcaacaagagcaa
	Reverse (5’-3’): atggtggtgctcgagctagaggttctccccgggctggtac
Src (K298M)	Forward (5’-3’): taccaccagggtggccatcaTGaccctgaagcctggc
	Reverse (5’-3’): atcgtgccaggcttcagggtCAtgatggccaccctgg
Src (Y530F)	Forward (5’-3’): cacgtccaccgagccccagtTccagcccggggagaa

	Reverse (5'-3'): agaggttctccccgggctggAactggggctcggtgg
FC-Src	Forward (5'-3'): gtggatccccggaattcatgggtagcaacaagagcaagcccaaggat
	Reverse (5'-3'): atggtggtgctcgagctagaggttctccccgggct
GST-Src	Forward (5'-3'): cgtggatccgaattcatgggtagcaacaagagcaagccca
	Reverse (5'-3'): gtggtggtgctcgagctagaggttctccccgggct
GFP-CSK	Forward (5'-3'): aagggatccgaattcatgtcagcaatacaggccgc
	Reverse (5'-3'): atggtggtgctcgagtcacaggtgcagctcgtgggttttg
GFP-Occludin	Forward (5'-3'): aagggatccgaattcatgtcatccaggcctcttgaaagtc
	Reverse (5'-3'): atggtggtgctcgagctatgttttctgtctatcatagtct

Supplementary Table 2. Primers for *Peak1*$^{-/-}$ mouse genotyping.

F1 (5'-3')	atggcaagcatctgaactgc
F2 (5'-3')	ggagtgagcctcctatattagcag
R (5'-3')	cattgcaaactgccaaggctc

Supplementary Table 3. Primers for qRT-PCR.

mouse. *Tjp1*	Forward (5'-3'): cccagcaatggcagtctccg
	Reverse (5'-3'): atgctgggcctaagtatcccg
mouse. *Ocln*	Forward (5'-3'): gcaaagtgaatggcaagcga
	Reverse (5'-3'): aggtggatattccctgaccca
human. *TJP1*	Forward (5'-3'): ccaagagcacagcaatggaggaa
	Reverse (5'-3'): ctccgttaaccattgcaactcgg
human. *OCLN*	Forward (5'-3'): aggcctgatgaattcaaaccga
	Reverse (5'-3'): aagttccatagcctctgtccc
18s	Forward (5'-3'): gtaacccgttgaaccccatt
	Reverse (5'-3'): ccatccaatcggtagtagcg
