# Supplementary material for: PEAK1 maintains tight junctions in intestinal epithelial cells and resists colitis by inhibiting autophagy-mediated ZO-1 degradation
Source: Nat Commun. 2025 Jul 24;16:6777. doi: 10.1038/s41467-025-62107-z (PMC12290104; doi:10.1038/s41467-025-62107-z)
Supplement: Supplementary file 2 — Reporting Summary [file 41467_2025_62107_MOESM2_ESM.pdf]

nature portfolio

Corresponding author(s): Yajun Xie, Magdalena Paolino, Qin Zhou

Last updated by author(s): Jul 7, 2025

Reporting Summary

Nature Portfolio wishes to improve the reproducibility of the work that we publish. This form provides structure for consistency and transparency in reporting. For further information on Nature Portfolio policies, see our Editorial Policies and the Editorial Policy Checklist.

Statistics

For all statistical analyses, confirm that the following items are present in the figure legend, table legend, main text, or Methods section.

n/a	Confirmed	
☐	☒	The exact sample size (*n*) for each experimental group/condition, given as a discrete number and unit of measurement
☐	☒	A statement on whether measurements were taken from distinct samples or whether the same sample was measured repeatedly
☐	☒	The statistical test(s) used AND whether they are one- or two-sided<br>*Only common tests should be described solely by name; describe more complex techniques in the Methods section.*
☒	☐	A description of all covariates tested
☐	☒	A description of any assumptions or corrections, such as tests of normality and adjustment for multiple comparisons
☐	☒	A full description of the statistical parameters including central tendency (e.g. means) or other basic estimates (e.g. regression coefficient) AND variation (e.g. standard deviation) or associated estimates of uncertainty (e.g. confidence intervals)
☐	☒	For null hypothesis testing, the test statistic (e.g. *F*, *t*, *r*) with confidence intervals, effect sizes, degrees of freedom and *P* value noted<br>*Give P values as exact values whenever suitable.*
☒	☐	For Bayesian analysis, information on the choice of priors and Markov chain Monte Carlo settings
☒	☐	For hierarchical and complex designs, identification of the appropriate level for tests and full reporting of outcomes
☒	☐	Estimates of effect sizes (e.g. Cohen's *d*, Pearson's *r*), indicating how they were calculated

Our web collection on statistics for biologists contains articles on many of the points above.

Software and code

Policy information about availability of computer code

Data collection	H&E and IHC were imaged by using Leica Appliation Suite X.3.7.2.22383 software. Immunofluorescence were imaged by using Leica Appliation Suite X 3.5.0.18371. FITC intensity were obtain by using SoftMax Pro 7.1. Transmission electron microscope were viewed using JEOL 1200EX II (JEOL, Peabody, MA) and photographed using a Gatan digital camera (Gatan, Pleasanton, CA). For LC-MS/MS Analysis, the peptide samples were separated by Nano liquid chromatography (EASY nLC-1200, Thermo Scientific), followed by Q Exactive HF-X mass spectrometer. Mass spectra were processed and searched using Proteome Discoverer (version 2.4, Thermo Scientific) against the human Swissprot protein database (release 2023_09).
Data analysis	Quantification of immunofluorescence data were analysed using fiji (NIH). Statistical analysis for in vitro and in vivo studies were conducted with GraphPad Prism software (Version 8.0.2) and OriginPro (Version 9.0.0). All figures were assembled using Adobe Illustrator 2022.

For manuscripts utilizing custom algorithms or software that are central to the research but not yet described in published literature, software must be made available to editors and reviewers. We strongly encourage code deposition in a community repository (e.g. GitHub). See the Nature Portfolio guidelines for submitting code & software for further information.

Data

Policy information about availability of data

All manuscripts must include a data availability statement. This statement should provide the following information, where applicable:

- Accession codes, unique identifiers, or web links for publicly available datasets
- A description of any restrictions on data availability
- For clinical datasets or third party data, please ensure that the statement adheres to our policy

The data, analytical methods, and study materials will be made available to other researchers. Further information and requests for resources and reagents should be directed to and will be fulfilled by the Lead Contact, Yajun Xie (yjxie@cqmu.edu.cn).

Research involving human participants, their data, or biological material

Policy information about studies with human participants or human data. See also policy information about sex, gender (identity/presentation), and sexual orientation and race, ethnicity and racism.

Reporting on sex and gender	N/A
Reporting on race, ethnicity, or other socially relevant groupings	N/A
Population characteristics	N/A
Recruitment	N/A
Ethics oversight	N/A

Note that full information on the approval of the study protocol must also be provided in the manuscript.

Field-specific reporting

Please select the one below that is the best fit for your research. If you are not sure, read the appropriate sections before making your selection.

[x] Life sciences [ ] Behavioural & social sciences [ ] Ecological, evolutionary & environmental sciences

For a reference copy of the document with all sections, see nature.com/documents/nr-reporting-summary-flat.pdf

Life sciences study design

All studies must disclose on these points even when the disclosure is negative.

Sample size	No statistical method was used to predetermine sample size in the study. All in vitro experiments were set up with at least three replicates. All in vivo experimental groups were performed with 5-12 mice/group to ensure reproducibility. These sample sizes also represent the standard practice for publication in this field and were described in figure legends. Each sample represents independent experimental replicates.
Data exclusions	There was no data excluded from the analysis.
Replication	In the construction of the DSS induced enteritis model in WT mice, a total of 20 mice in the DSS treatment group were selected for detection according to the time gradient, and 3 mice in the control group were randomly selected for detection. To detect intestinal permeability in vivo, a total of 6 replicates were performed in WT mice and 5 replicates were performed in KO mice. When the enteritis model was constructed using PEAK1 knockout mice, 12 mice in each experimental group and control group were repeated. The replication of other experiments are described in figure legend.
Randomization	N/A
Blinding	The investigator could not be blinded as they were responsible for both the preparation of the samples and analysis of the data.

Reporting for specific materials, systems and methods

We require information from authors about some types of materials, experimental systems and methods used in many studies. Here, indicate whether each material, system or method listed is relevant to your study. If you are not sure if a list item applies to your research, read the appropriate section before selecting a response.

Materials & experimental systems

n/a	Involved in the study	
☐	☐	Antibodies
☐	☒	Eukaryotic cell lines
☒	☐	Palaeontology and archaeology
☐	☒	Animals and other organisms
☒	☐	Clinical data
☒	☐	Dual use research of concern
☒	☐	Plants

Methods

n/a	Involved in the study	
☒	☐	ChIP-seq
☒	☐	Flow cytometry
☒	☐	MRI-based neuroimaging

Antibodies

Antibodies used

1. PEAK1 (Novus Biologicals, Cat# NBP1-91052)
2. PEAK1 (Cell Signaling Technology, Cat# 72908)
3. ZO-1 (Invitrogen, Cat# 33-9100)
4. ZO-1 (Proteintech, Cat# 21773-1-AP)
5. Src (Cell Signaling Technology, Cat# 2108)
6. Phospho-Src (Tyr416) (Cell Signaling Technology, Cat# 2101)
7. Phospho-Src (Y527) (Cell Signaling Technology, Cat# 2105)
8. Occludin (Bioworlde, Cat# AP0765)
9. Occludin (Abcam Cat# ab216327)
10. CSK (Proteintech, Cat# 17720-1-AP)
11. LC-3B (Cell Signaling Technology, Cat# 2775)
12. LC-3 (MBL, Cat# 4E12)
13. anti-GFP (Abcam, Cat# ab290)
14. anti-GFP (Bioworlde, Cat# MB 9233)
15. anti-FC (Abcam, Cat# ab190492)
16. ERK1/2 (Cell Signaling Technology, Cat# 4695)
17. β-actin (Transgen, Cat# HC201-01)
18. β-Tubulin (Transgen, Cat# HC101-01)
19. GAPDH (Abclonal, Cat# AC002)
20. anti-Flag (Abclonal, Cat# AE005)
21. anti-Phosphotyrosine (4G10) (Millipore, Cat# 05-321)
22. Phospho-PEAK1 (Y724) (This study, N/A)
23. Anti-Mouse IgG, HRP (CWBIO, Cat# CW0102)
24. Anti-Rabbit IgG, HRP (CWBIO, Cat# CW0103)
25. Conformation Specific Mouse Anti-Rabbit IgG (Cell Signaling Technology, Cat#5127S)
26. Goat anti-Mouse IgG, Alexa Fluor® 488 conjugate (Invitrogen, Cat# A-11029)
27. Goat anti-Rabbit IgG, Alexa Fluor® 488 conjugate (Invitrogen, Cat# A-11034)
28. Goat anti-Mouse IgG, Alexa Fluor® 555 conjugate (Invitrogen, Cat# A-21424)
29. Goat anti-Rabbit IgG, Alexa Fluor® 555 conjugate (Invitrogen, Cat# A-21429)
30. Phospho-Myosin Light Chain 2 (Ser19) (Cell Signaling Technology, Cat#3671)
31. Myosin Light Chain 2 (Cell Signaling Technology, Cat#8505)

Validation

Antibodies were selected in accordance with prior publications and the manufacturer's recommendations (based on their website data). All antibodies successfully detected proteins of their expected molecular weights. p-PEAK1 (Y724) antibody in this study was validated by IP and western blot.

Eukaryotic cell lines

Policy information about cell lines and Sex and Gender in Research

Cell line source(s)	Caco-2 (SCSP-5027), HEK293T (SCSP-502) and MDCK (SCSP-5258) were purchase from National Collection of Authenticated Cell Cultures (https://www.cellbank.org.cn).<br>NCM460 (BFN608006385) was purchase from BFB Corporation (http://www.bluefcell.com/).
Authentication	Provider's authenticated cell lines, and we also assessed cell morphology by microscopy.
Mycoplasma contamination	Cell lines were routinely screened for mycoplasma contamination using the mycoplasma detection kit.
Commonly misidentified lines (See ICLAC register)	N/A

Animals and other research organisms

Policy information about studies involving animals; ARRIVE guidelines recommended for reporting animal research, and Sex and Gender in Research

Laboratory animals	Peak1 knockout C57BL/6J mice were designed and constructed by Biocytogen company.
Wild animals	N/A
Reporting on sex	According to existing literature, only male subjects were used in our experiment.<br>1. Yanlei Ma. et al. ACF7 regulates inflammatory colitis and intestinal wound response by orchestrating tight junction dynamics. Nat Commun 2017 Jul 11;8(0):16121<br>2. Sujin Kang. et al. TRIM40 is a pathogenic driver of inflammatory bowel disease subverting intestinal barrier integrity. Nat Commun 2023 Feb 9;14(1):700
Field-collected samples	No field collected samples were used in the study.
Ethics oversight	All mouse studies were approved by the Institutional Animal Care and Use Committee (IACUC) of Chongqing Medical University. The mice were housed in specific pathogen-free conditions at the animal center of Chongqing Medical University and were subject to a strict 12-hour light-dark cycle (lights on at 8 am and off at 8 pm). They were accommodated in cages with a maximum occupancy of five animals.

Note that full information on the approval of the study protocol must also be provided in the manuscript.

Plants

Seed stocks	N/A
Novel plant genotypes	N/A
Authentication	*Describe any authentication procedures for each seed stock used or novel genotype generated. Describe any experiments used to assess the effect of a mutation and, where applicable, how potential secondary effects (e.g. second site T-DNA insertions, mosiacism, off-target gene editing) were examined.*
